# Public Knowledge, Perception and Source of Information on Ebola Virus Disease – Lagos, Nigeria; September, 2014

**DOI:** 10.1371/currents.outbreaks.0b805cac244d700a47d6a3713ef2d6db

**Published:** 2015-04-08

**Authors:** Saheed Gidado, Abisola M. Oladimeji, Alero Ann Roberts, Patrick Nguku, Iruoma Genevieve Nwangwu, Ndadilnasiya Endie Waziri, Faisal Shuaib, Olukayode Oguntimehin, Emmanuel Musa, Charles Nzuki, Abdulsalami Nasidi, Peter Adewuyi, Tom-Aba Daniel, Adebola Olayinka, Oladoyin Odubanjo, Gabriele Poggensee

**Affiliations:** Nigeria Field Epidemiology and Laboratory Training Programme, Abuja, Nigeria; Nigeria Field Epidemiology and Laboratory Training Programme, Abuja, Nigeria; Department of Community Health & Primary Care, College of Medicine, University of Lagos, Nigeria; Nigeria Field Epidemiology and Laboratory Training Programme, Abuja, Nigeria; Department of Community Health, Lagos University Teaching Hospital, Idi-Araba, Lagos, Nigeria; Nigeria Field Epidemiology and Laboratory Training Programme, Abuja, Nigeria; Federal Ministry of Health, Abuja, Nigeria; Lagos State Primary Health Care Board, Lagos, Nigeria; World Health Organization, Abuja, Nigeria; United Nations Children’s Fund, Enugu, Nigeria; Nigeria Centers for Disease Control, Abuja, Nigeria; Nigeria Field Epidemiology and Laboratory Training Programme, Abuja, Nigeria; Nigeria Field Epidemiology and Laboratory Training Programme, Abuja, Nigeria; Nigeria Field Epidemiology and Laboratory Training Programme, Abuja, Nigeria; Nigerian Academy of Science, University of Lagos, Nigeria; Nigeria Field Epidemiology and Laboratory Training Programme, Abuja, Nigeria

**Keywords:** ebola, ebolavirus

## Abstract

Background: The first ever outbreak of Ebola virus disease (EVD) in Nigeria was declared in July, 2014. Level of public knowledge, perception and adequacy of information on EVD were unknown. We assessed the public preparedness level to adopt disease preventive behavior which is premised on appropriate knowledge, perception and adequate information. Methods: We enrolled 5,322 respondents in a community-based cross-sectional study. We used interviewer-administered questionnaire to collect data on socio-demographic characteristics, EVD–related knowledge, perception and source of information. We performed univariate and bivariate data analysis using Epi-Info software setting p-value of 0.05 as cut-off for statistical significance. Results: Mean age of respondents was 34 years (± 11.4 years), 52.3% were males. Forty one percent possessed satisfactory general knowledge; 44% and 43.1% possessed satisfactory knowledge on mode of spread and preventive measures, respectively. Residing in EVD cases districts, male respondents and possessing at least secondary education were positively associated with satisfactory general knowledge (p-value: 0.01, 0.001 and 0.000004, respectively). Seventy one percent perceived EVD as a public health problem while 61% believed they cannot contract the disease. Sixty two percent and 64% of respondents will not shake hands and hug a successfully treated EVD patient respectively. Only 2.2% of respondents practice good hand-washing practice. Television (68.8%) and radio (55.0%) are the most common sources of information on EVD. Conclusions: Gaps in EVD-related knowledge and perception exist. Targeted public health messages to raise knowledge level, correct misconception and discourage stigmatization should be widely disseminated, with television and radio as media of choice.

## Introduction

Ebola viral disease (EVD) is a severe, often fatal illness, with a case fatality rate of up to 90% if untreated.^1^ The virus is transmitted to people from wild animals and spreads in communities through human-to-human transmission.The disease was first discovered in 1976 in two simultaneous outbreaks in Sudan and Democratic Republic of Congo. The current outbreak in West Africa began in Guinea in 2013.^2^ It is the largest and most complex EVD outbreak since the virus was first discovered.^1^^,^ 3 By 10^th^October, 2014, a total of 8397 cases with 4032 deaths were recorded in five West-African countries namely Guinea, Liberia and Sierra Leone, Nigeria and Senegal. The highest reported case counts are from Liberia (4076 cases), Sierra Leone (2950 cases) and Guinea (1350 cases).^4^

The first EVD case in Nigeria was recorded on 20^th^ July, 2014 in an acutely ill traveller from Liberia.^5^ Prior to the current outbreak, Nigeria has not had an occurrence of the disease; hence the scenario created public fear, panic and confusion, as is usually seen in outbreaks of previously unknown diseases.^6^ Furthermore, there was paucity of locally relevant information to guide the key public health measures, implemented to prevent community spread of the disease. The attendant fear associated with an outbreak of EVD is capable of undermining the outbreak control efforts.^7^

Following the declaration of the outbreak, the Federal Ministry of Health /Nigerian Centers for Disease control (FMOH/NCDC) in collaboration with the Lagos State Ministry of Health and partner agencies established an Ebola Emergency Operations Centre (EEOC) to coordinate all outbreak response activities. 5 The social mobilization and communication team of the EEOC conducted targeted, house-to-house EVD education activities in the areas where cases and contacts of people with EVD lived. These activities were commenced about one week before the implementation of this survey. Additionally, widespread campaigns were implemented in the state on mass and social media platforms providing health promotion messages that aimed to address public concerns and promote adoption of EVD preventive/risk reduction behaviour to reduce community transmission. A toll-free EbolaHelp phone number was established und published which is open for the public to ask questions concerning EVD and to seek medical advice when there was concerns of EVD. However, the influence of these messages on the level of knowledge, attitude and behaviour are not known.

## Methods

The survey was conducted in Lagos State, south-west Nigeria, with a population of about 17.5 million.^8^ Administratively, Lagos state has 20 Local Government Areas (LGAs).^9^ Lagos is a highly heterogeneous state, comprising ethnic groups from virtually all over the country and home to significant international populations. There are 379 wards spread across these 20 LGAs, with 276 Primary Health Care Centers (PHCC) which serve as the first point of contact for citizens seeking health care services.

We conducted a cross-sectional study; the respondents were individuals aged 18 years and above who live or trade in communities studied. For any respondent to be eligible for recruitment for the survey, (s)he must have lived in the area for not less than four weeks. Sample size was determined using Cochran’s formula.

Using multi-stage sampling, twelve LGAs were selected proportionately from the 3 senatorial zones. Six LGAs were selected from Lagos West and 3 each from Lagos East and Lagos Central respectively. Wards were proportionately sampled from the selected LGAs; 40, 20 and 18 wards were randomly selected from the LGAs in Lagos West, Lagos East and Lagos Central zones respectively.To select the communities for the study, we created the map of the 78 wards using Google mapping to identify the streets. Each ward was divided into four quadrants. Beginning from the right upper quadrant and moving in a clock-wise direction, one-third of the streets in the quadrant were randomly selected. In selected streets interviewers visited every other house starting from the first house on the right. One respondent who met the inclusion criteria was randomly selected from each house by balloting. In multi-dwelling houses, one household was randomly selected and one respondent selected from the household. A minimum of 17 respondents were selected per quadrant to ensure a minimum sample size of 66 respondents per ward.n=z^2^pq/d^2^assuming* a*prevalence of knowledge or practice of 50%* (in the *absence of any documented prevalence of community knowledge on EVD), precision level of 5%. Correcting for 10% non-response rate, we calculated the minimum sample size per local government area to be 423 respondents.^10^ We sampled respondents from 12 out of the 20 LGAs in the state, making the total sample size 5,076.

Data were collected using semi-structured, interviewer-administered, paper-based questionnaires on socio-demographic characteristics, active knowledge on EVD, perception, behavioral practices and sources of information. Questionnaires were retrieved daily, and reviewed to exclude incomplete forms. Data were entered and analyzed using Epi-Info 3.5.4 and reported as frequencies and percentages. Associations between variables were tested statistically using Chi-square and reported at a significance level of p< 0.05. Knowledge was graded based on three EVD domains; mode of spread, symptoms and signs, and preventive measures. Weighted scores were assigned to correct responses mentioned by respondents. Respondents who scored a total of 10 points and above were considered to possess satisfactory general knowledge.^11^^,^ 12 Stratified analysis was done among those with satisfactory knowledge to assess knowledge across each domain.

The study was conducted by the Epidemiology and Suveillance Team/Operational Research of the national Ebola Emergency Operation Center (EEOC) in Lagos as part of the response to the EVD outbreak in Lagos State. The senior strategy group of the EEOC responsible for the overall design of the response reviewed and approved the study^5^. Informed consent was obtained from the respondents who were assured of voluntary participation, confidentiality of their responses and the opportunity to withdraw at any time without prejudice in line with the Helsinki Declaration.^13^

## Results

A total of 5,322 respondents were interviewed, 52.3% were males; the mean age was 34 years (+ 11.4 years). The respondents were largely Christian (70.4%), with secondary or post-secondary education (84.5%) and were either artisans or traders (60.3%) (Table 1).

**Table 1: Socio-demographic characteristics of respondents:** 

Socio-demographic characteristics (N=5322)	Number of respondents (%)
**Age distribution (in years)**	
≤ 20	503 (9.5)
21 – 30	1946 (36.6)
31 – 40	1563 (29.4)
41 – 50	815 (15.3)
> 50	495 (9.3)
**Sex**	
Male	2785 (52.3)
Female	2537 (47.7)
**Religion**	
Christianity	3747 (70.4)
Islam	1507 (28.3)
Traditional	46 (0.9)
Others	22 (0.4)
**Highest level of formal education**	
None	145 (2.7)
Primary	680 (12.8)
Secondary	2826 (53.1)
Post-secondary (Tertiary)	1671 (31.4)
**Occupation**	
Traders/Business	2021 (38.0)
Artisans	1185 (22.3)
Unemployed/Housewives/Students	833 (15.7)
Professionals/Civil servants	627 (11.8)
Others	413 (7.8)
Drivers	113 (2.1)
Missing	95 (1.8)
Clergy	35 (0.7)

Thirty three percent of respondents do not know the cause of EVD, 17%, 11% and 6% of respondents mentioned non-human primates, bush meat and bats as the causes of EVD, respectively. About 0.4% of respondents mentioned that EVD is caused by the Liberian traveller, while 0.3% affirmed that the disease is caused by western world. The three commonest modes of spread of EVD mentioned by the respondents were contact with a person who is sick of EVD (69.4%), touching body fluids of a person who is sick of EVD (47.3%), and contact between infected animals and men (33.4%). Sixteen percent of respondents mentioned contact with clothing, beddings and other utensils of a person who is sick of EVD while approximately 6% mentioned participation in the burial rites of a person who died of EVD as possible modes of spread of the disease. The top three signs and symptoms of EVD mentioned by respondents were fever (56.9%), vomiting (48.3%) and abnormal bleeding (38.1%) (Figure 1). Of the various EVD preventive measures 66% and 49% of respondents mentioned regular hand washing with soap and water, and avoiding contact with EVD case or suspect, respectively. Sixteen percent mentioned avoiding eating bush meat while 5% mentioned not participating in the burial rite of a person who died of EVD.

**Signs and symptoms of EVD mentioned by respondents:**
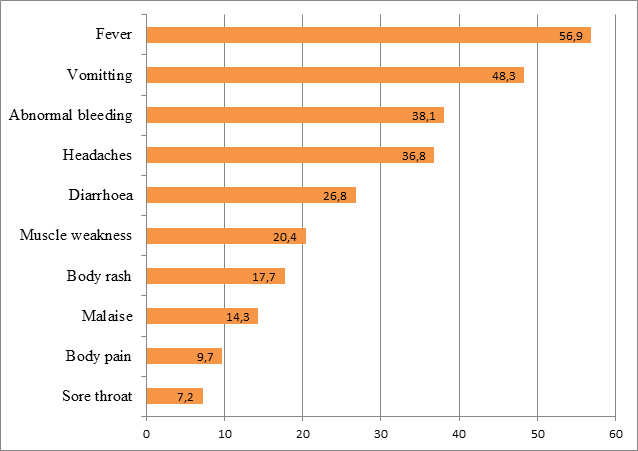

Eighteen percent of respondents indicated that there is either a specific drug or specific remedy to treat EVD, while 7% affirmed that there is a specific vaccine to prevent the disease. Some of the drugs mentioned included two widely publicized EVD trial drugs namely Zmapp and Nano-silver (Nigeria only). Some specific remedies mentioned by respondents to treat EVD included drinking and bathing with salt-water solution, consumption of local medicinal herbs and eating bitter kola (also known as *Garcinia kola* – a specie of the flowering plant in the Clusiaceae or Guttiferae family found in large quantities in West Africa) – all of which were widely rumoured in Nigeria during the EVD outbreak as effective interventions to treat EVD. A few respondents mentioned fervent prayers, fasting and other spiritual interventions to treat EVD. Of the 5,322 respondents, 41.1% possessed satisfactory general knowledge while 18.7% possessed satisfactory knowledge in all three domains. Seventy eight percent, 44% and 43.1% possessed satisfactory knowledge in signs and symptoms, preventive measures and mode of spread respectively (Table 2).

**Table 2: Knowledge score of respondents on EVD across all domains:** 

	Respondents with satisfactory knowledge across all domains				
LGA	Mode of spread	Symptoms and signs	Preventive measures	General knowledge	Knowledge in all domains
Ajeromi (n=345)	138 (40)	276 (80)	143 (41.5)	135 (39.1)	54 (15.7)
Alimosho (n=844)	353 (41.8)	658 (78)	395 (46.8)	358 (42.4)	178 (21.1)
Amuwo Odofin (n=374)	166 (44.4)	287 (76.7)	150 (40.1)	139 (37.1)	53 (14.2)
Apapa (n=279)	137 (49.1)	214 (76.7)	118 (42.3)	117 (41.9)	67 (24.0)
Ibeju Lekki (n=276)	151 (54.7)	212 (76.8)	112 (40.6)	122 (44.2)	39 (14.2)
Ifako Ijaiye (n=358)	164 (45.8)	275 (76.8)	155 (43.3)	157 (43.9)	85 (23.7)
Ikorodu (n=677)	252 (37.2)	522 (77.1)	275 (40.6)	264 (39.0)	106 (15.7)
Lagos Mainland (n=360)	151 (41.9)	282 (78.3)	165 (45.8)	145 (40.3)	68 (18.9)
Mushin (n=336)	155 (46.1)	254 (75.6)	190 (56.6)	126 (37.5)	69 (20.5)
Oshodi Isolo (n=538)	235 (43.7)	413 (76.8)	249 (46.3)	246 (45.7)	106 (19.7)
Shomolu (n=337)	106 (31.5)	272 (80.7)	161 (47.8)	132 (39.2)	45 (13.4)
Surulere (n=598)	284 (47.5)	474 (79.3)	273 (45.7)	260 (43.5)	109 (18.2)
All respondents n=5322)	2294 (43.1)	4141 (77.8)	2342 (44.0)	2203 (41.4)	995 (18.7)

Respondents who possessed poor knowledge on mode of spread were more likely to possess poor knowledge on preventive measures (p-value: 0.001). Respondents who reside in LGAs where EVD cases were recorded, male respondents and respondents with at least secondary education were more likely to possess satisfactory general knowledge (p-value: 0.01, 0.001 and 0.000004 respectively). There was no association between residing in LGAs where social mobilization activities have taken place and satisfactory knowledge. (Table 3)

**Table 3: Association between level of knowledge on EVD and key characteristics:** 

Variable	Poor knowledge	Satisfactory knowledge	Odds Ratio(95% CI)	p-value
**Gender**				
Female	1558 (61.4)	979 (38.6)	1×2 (1×1 - 1×4)	0×0001
Male	1563 (56.1)	1222 (43.9)		
**Educational level**				
Low (None and Primary)	545 (66.0)	280 (33.9)	1.5 (1.2 - 1.7)	0×000004
High (Secondary and Tertiary)	2576 (57.3)	1921 (42.7)		
**LGA type**				
Rural	567 (59.5)	386 (40.5)	1.04 (0.9 - 1.2)	0×56
Urban	2554 (58.5)	1815 (41.5)		
SMC LGAs*				
No	1711 (59.9)	1145 (40.1)	1×1 (1×0 - 1×2)	0×05
Yes	1410 (57×2)	1056 (42×8)		
**LGAs with EVD cases**				
No	2491 (59.5)	1695 (40×5)	1×2 (1×0 - 1×3)	0×014
Yes	630 (55×5)	506 (44×5)		
**Hand-washing practice**				
Poor	3074(59×1)	2129(40×9)	2×2 (1×5 - 3×2)	0×000027
Good	47(39×5)	72(60×5)		
**EVD care-seeking behavior**				
Will not go hospital	862(59×4)	589(40×6)	1×04 (0×9 - 1×2)	0×51
Will go to hospital	2259(58×4)	1612(41×6)		

Seventy one percent of respondents perceived EVD as a problem in Lagos State, 61% felt that they cannot contract EVD. Majority of these respondents mentioned spiritual and divine protection, and observing appropriate precautions as the reasons for this perception. About 76% of respondents thought that the government is doing enough to contain the EVD outbreak, 13% thought otherwise while 11% maintained a neutral position. Respondents who thought the government is not doing enough opined that government should have found the drug and/or vaccine for the disease, closed the country’s international borders, prevented foreigners from entering the country, scaled-up social mobilization activities and provided free sanitizers to the public. Seventy three percent of respondents mentioned that they will go to a health facility if they developed EVD-like signs and symptoms; 16% would call the Ebola Alert number. Seventy two percent of respondents would advise someone with EVD-like symptoms to go to health facility, 18.5% would advise calling the Ebola Alert number. Asked to demonstrate handwashing, respondents washed palms only (87%), back of hands (83%) and fingers (38%). Only 119 respondents (2.2%) washed all parts of their hands. Possession of satisfactory knowledge of spread and prevention by use of handwashing with soap and water was associated with use of correct handwashing techniques (p = 0.00003 and 0.004, respectively). A total of 2162 (41%) respondents had stopped engaging in activities such as shaking hands, hugging, eating bush meat and unnecessary contact with people since the onset of the EVD in Lagos. Of those that have not stopped these activities, 62% will not shake hands with persons who have recovered from EVD; 64% will not hug such persons. There was no association between their decision and their knowledge level of respondents. However, 3084 (58%) respondents stated that they started regular hand washing, keeping the environment clean and maintaining a higher standard of personal hygiene. A few respondents (6%) reported bathing with salt and water and eating kola nut to protect themselves. In all the LGAs, television and radio were the most common sources of information on EVD. Generally, 69% and 55% of respondents mentioned television and radio, respectively, as their sources of information on EVD.

Twelve percent and 9% of respondents got information on EVD through the internet and social media respectively. Altogether, 37% had heard of the Ebola helpline, only 36/1993 (1.8%) got the number correct. Furthermore, 16% had heard of the www.ebolaalert.com website, 12.3% got the correct URL.

## Discussion

The importation of EVD into Lagos state underscored the risk of urban spread of the disease. Findings from this study revealed the disparate levels of knowledge which can prevent further disease transmission. EVD has not previously been known to occur in Nigeria, therefore there have been no previous studies conducted on community preparedness. Literature review also revealed a paucity of published data in Nigeria. Knowledge about mode of spread of EVD was generally low. 14 ^,^ 15 ^,^ 16 Findings indicate that community members with good knowledge on how EVD is spread, are more likely to adopt appropriate measures to prevent community spread.^14^ More respondents knew animal to man as a mode of spread, which is not as important as contact with fomites or participating in burial of a person who died of EVD.^1.5^ Certain cultural rites performed widely in many African countries, before burying a corpse encourages community spread of the Ebola virus.^16^ These findings provide a rationale to modify the communication messages as part of the ongoing social mobilization efforts to contain the disease.

The government’s effort to contain the EVD outbreak in Nigeria has been widely commended at both national and international levels.^5^ This was corroborated by three out of every four respondents who believed that government is doing enough to contain the EVD outbreak. Despite the known value of proper handwashing techniques, it has not translated to wide practice. 17 This informs the need for teaching proper hand-washing as a means of preventing spread of highly infectious diseases.^18^

At the beginning of the outbreak in Nigeria, television and radio stations aired different public enlightenment programs on EVD as part of their “social cooperate responsibilities”. Television and radio should be the target to disseminate public health information on EVD and other diseases in future outbreaks. Stigmatization of recovered EVD patients was prominent and has implications for EVD prevention and control.^19^^,^ 20 Failure to promptly identify EVD cases impedes implementation of key public health preventive measures and enhances community spread of the disease. Deriving evidence from this finding, a major communication focus for the social mobilization group is to intensify public health education with respect to the “safe status” of a person who has been treated of EVD while mounting an aggressive campaign against stigmatization of EVD cases, suspects and contacts.

Our survey indicated that despite the implementation of social mobilization activities in some LGAs, level of EVD knowledge among residents in these LGAs did not differ from those of resident in other LGAs. Findings in this survey could serve as baseline, with repeat surveys after 4 – 6 weeks in the same LGAs to assess the impact of social mobilization.

A major challenge we encountered during the survey was shortage of reliable data entry clerks. Providing daily updates to Ebola EOC required daily data entry and prompt data analysis. Based on this challenge, we recommend the use of real-time electronic data collection using open data kit (ODK) for future large scale operational research.

## Conclusions

The survey surpassed mere academic exercise by providing evidenced-based information that guided the implementation of social mobilization activities and dissemination of appropriate public health information as part of the EVD response in Nigeria. We recommend development of health messages focusing on the mode of spread and preventive measures, demonstration of hand-washing techniques and social mobilization campaigns to prevent stigmatization of EVD cases and contacts. Radio and television should be used to disseminate relevant accurate health information to the public. A repeat survey should be done to monitor changes in knowledge and behavior. Finally, this survey highlights the need for real time data gathering as part of an outbreak response.

## Supporting Information

Because outbreak of Ebola virus disease has not been previously recorded in Nigeria, there is little or no information on the level of public knowledge, perception, practices and source of information regarding the disease. This study was conducted as part of the outbreak response, to assess the level of public preparedness to adopt risk reduction behavior which is premised on appropriate knowledge and perception. The study provided information on the level of public knowledge, perception and risk reduction behavior which were previously unknown in the study area. Information obtained from the study guided the strategy and content of health communication messages during the outbreak which contributed to the overall response and containment of the outbreak.

## Correspondence

Gabriele Poggensee: gapo.nigeria@gmail.com
